# Expression of Duodenal Iron Transporter Proteins in Diabetic Patients with and without Iron Deficiency Anemia

**DOI:** 10.1155/2018/7494821

**Published:** 2018-03-06

**Authors:** Efrat Broide, Ram Reifen, Shay Matalon, Zipi Berkovich, Haim Shirin

**Affiliations:** ^1^The Kamila Gonczarowski Institute of Gastroenterology, Assaf Harofeh Medical Center and Sackler School of Medicine, Tel Aviv University, Tel Aviv, Israel; ^2^School of Nutritional Sciences, Faculty of Agriculture, The Hebrew University of Jerusalem, Jerusalem, Israel

## Abstract

The role of iron transport proteins in the pathogenesis of anemia in patients with diabetes mellitus (T2DM) is still unclear. We investigated the expression of duodenal transporter proteins in diabetic patients with and without iron deficiency anemia (IDA). *Methods*. Overall, 39 patients were included: 16 with T2DM and IDA (group A), 11 with T2DM without IDA (group B), and 12 controls (group C). Duodenal mucosal expression of divalent metal transporter 1 (DMT1), ferroportin 1 (FPN), hephaestin (HEPH), and transferrin receptor 1 (TfR) was evaluated by Western blotting. Chronic disease activity markers were measured as well. *Results*. FPN expression was increased in group A compared to group B and controls: 1.17 (0.72–1.46), 0.76 (0.53–1.04), and 0.71 (0.64–0.86), respectively (*p* = 0.011). TfR levels were over expressed in groups A and B compared to controls: 0.39 (0.26–0.61), 0.36 (0.24–0.43), and 0.18 (0.16–0.24), respectively, (*p* = 0.004). The three groups did not differ significantly with regard to cellular HEPH and DMT1 expression. The normal CRP and serum ferritin levels, accompanied with normal FPN among diabetic patients without IDA, do not support the association of IDA with chronic inflammatory state. *Conclusion*. In patients with T2DM and IDA, duodenal iron transport protein expression might be dependent on body iron stores rather than by chronic inflammation or diabetes per se.

## 1. Introduction

Iron absorption by the proximal small bowel is a critical checkpoint in the maintenance of whole-body iron level, as no regulated excretory systems exist for iron in humans [1]. Ferritin has been proposed to be a component of insulin resistance syndrome, as higher heme iron intake and ferritin are associated with a greater risk of type 2 diabetes mellitus (T2DM) [2]. The pathogenesis is most probably the susceptibility of *β* cells to iron deposition leading to insulin deficiency. By contrast, the estimated prevalence of anemia in diabetic patients is about 10% to 30% [3], while approximately one-third exhibit iron deficiency. Failure to produce erythropoietin in response to declining hemoglobin is a common contributor to the anemia and could well be a manifestation of diabetic kidney disease, regardless of renal impairment [4].

Any inflammatory conditions are often associated with significant changes in systemic iron metabolism, with altered metabolic state as there is a decrease in plasma iron levels due to reduced intestinal absorption and increased intracellular sequestration of the metal [5]. T2DM, one of the most prevalent chronic inflammatory diseases worldwide, is associated with cytokine secretion that simultaneously increases transferrin receptors (TfR) on the cell surface. The inflammatory cytokines induce hepcidine (HEPC) overproduction which causes the endocytosis and proteolysis of ferroportin (FPN), favoring trapping iron in the absorbing enterocytes resulting in iron deficiency anemia (IDA) [6].

This study aimed to further elucidate the mechanism of IDA in T2DM patients by exploring the involvement of iron absorption and iron transportation proteins in the cellular level of the duodenum.

## 2. Patients and Methods

This case-control study included 27 patients with T2DM for at least 5 years duration. Patients were divided into 2 groups according to their hemoglobin and iron status: group A, T2DM patients with IDA (*n* = 16); group B, T2DM without IDA (*n* = 11). Twelve patients without anemia nor T2DM served as controls (group C). Only patients who needed endoscopy due to clinical indications were included. All patients had normal kidney function (creatinine clearance > 60 ml/min). Patients with known chronic inflammatory disorders were excluded. During the upper endoscopy, three biopsies were taken; two from the stomach (corpus and antrum) for evaluation of gastritis and *Helicobacter pylori* infection and a single duodenal biopsy to exclude celiac disease. Additional two duodenal biopsies were taken to investigate the iron metabolism and homeostasis in the enterocytes. Venous blood was drawn to evaluate hemoglobin levels, CRP, iron, ferritin, transferrin and transferrin saturation, glucose, and HbA1C. Anemia was defined as hemoglobin values < 13 g/dL for men and <12 g/dL for women. All participants filled a questionnaire which included demographic and clinical data, drug history, duration of T2DM, and accompanying comorbidities. This study was approved by the local ethics committee.

### 2.1. Western Blotting

Protein extracts were prepared from nitrogen frozen duodenal biopsy specimens using MUNRO lysis buffer (10 mM HEPES, 3 mM MgCl2, 40 mM KCl, 5% (*w\v*) glycerol, and 0.2% NP40/Igepal), and protein concentrations were determined by BCA assay (Bio-Rad Laboratories). 100 *μ*g of total protein was electrophoresed on a 15% SDS-polyacrylamide gel and was transferred to a polyvinylidene fluoride membrane (MilliPore) preincubated with blocking solution (5% *w/v* nonfat dry milk in Tris-buffered saline containing 0.05% Tween 20, pH 7.4) for 20 min at room temperature, overnight incubation at 4°C with affinity-purified rabbit antihuman FPN antibody (NBP1-21502, Novus Biological), affinity-purified rabbit antihuman TfR (NBP1-95317, Novus Biological), rabbit antihuman DMT1/NRAMP (sc-30120, ENCO), mouse antihuman heavy (H) ferritin (ab-77127, Abcam), mouse antihuman light (L) ferritin (sc-74513, Santa Cruz Biotechnology), and mouse antihuman hephaestin (HEPH) (sc-365365, Santa Cruz Biotechnology), using dilutions of 1 : 1000, 1 : 5000, 1 : 500, 1 : 500, 1 : 250, and 1 : 500, respectively. As a control, *β*-actin was loaded simultaneously with the other antibodies and used to normalize protein expression (1 : 500 dilution of rabbit antihuman *β*-actin antibody (ab52614, Epitomics)). Secondary antibody (HRP-linked goat anti-rabbit/anti-mouse IgG antibody, Jackson ImmunoResearch) was used at 1 : 10,000 dilution. Molecular weight standards (Precision Plus Protein Standards, Bio-Rad Laboratories) were run in parallel. Immunoreactivity was detected by chemiluminescent HRP substrate (Western Bright ECL, Advansta) and quantified using Imagelab software.

### 2.2. Sample Size

Calculation of the needed patients in the diabetic groups (with power of 90% and significant level of 5%), assuming that the mean difference of TfR level is of 0.1 with a SD of 0.05, revealed 12 needed patients in each group.

### 2.3. Statistical Analysis

Categorical variables are presented as number and percentage. Continuous variables were evaluated for normal distribution using histogram and Q-Q plot. All the continuous variables are presented as median and interquartile range (IQR). Categorical variables were compared between groups using chi square test or Fisher's Exact Test as appropriate. Continuous variables were compared between groups using analysis of variance (ANOVA) or Kruskal-Wallis test. Post hoc analysis was performed after test for the homogeneity of variances. LSD or Tamhane's T2 was used after ANOVA and Mann–Whitney test after Kruskal-Wallis test.

All statistical tests were two sided, and *p* value < 0.05 was considered as statistical significant. All statistical tests were performed using SPSS version 24 (IBM SPSS Statistics, IBM Corp., Armonk, NY., USA, 2016).

## 3. Results

As expected, the mean hemoglobin level in group A was 10.9 (IQR 10.4–12.2) compared to 13 (IQR 12.5–14.3) in group B and 14.1 (IQR 13.1–14.6) in group C. Diabetic patients, with or without IDA, had more comorbidities and used more frequently aspirin and proton pump inhibitors. Group A patients were significantly older than the other two groups (*p* < 0.05). No significant differences were found in HbA1C% between patients with T2DM with or without IDA. No significant differences were found in CRP levels between groups (Table 1).

As shown in Table 2, FPN expression in the duodenum was increased significantly in patients with T2DM with anemia compared to patients without IDA and controls; 1.17 (0.89–1.46), 0.76 (0.53–1.04), and 0.71 (0.64–0.86), respectively (*p* = 0.011). TfR levels were over expressed in both groups with diabetes compared to the controls: 0.39 (0.26–0.61), 0.36 (0.24–0.43), and 0.18 (0.16–0.24), respectively (*p* = 0.004). The three groups did not differ significantly with regard to cellular HEPH and divalent metal transporter 1 (DMT1) expression. Stepwise logistic regression analysis revealed that IDA in T2DM patients was positively associated with older age: OR 1.03 (*p* < 0.03), increasing the risk for IDA by 3% every coming year.

## 4. Discussion

Diabetic complications are mainly related to oxidative stress and ischemic damage [7]. Anemia with iron deficiency increases the lipid peroxidation and HbA1C levels, suggesting the increase in oxidative stress may significantly increase the risk of late-stage complications. Inflammation and infection induce HEPC expression, likely explaining the hypoferremia that accompanies chronic inflammatory conditions [6]. Our results indicate that TfR and FPN expression in the duodenum is over expressed in T2DM patients with IDA. These results are consistent with previous reports that both intestinal proteins are strongly upregulated in IDA [1].

TfR is expressed on all dividing cells, and its rate of synthesis is closely linked to the requirements of the cell for iron. It has been suggested that TfR1-mediated iron uptake from the basolateral side of the intestinal crypt cells determines the level of the expression of iron transport proteins [8]. FPN, also known as Slc40a1, is the only known iron exporter in mammals. It is expressed in cells that absorb (enterocytes) and store (hepatocytes and reticuloendothelial macrophages) iron. Iron that reaches the cytosol is then pumped out, either at the basolateral surface of the enterocyte or the plasma membrane of the macrophage, by FPN [5].

Because the transmembrane proteins, DMT1 and HEPH, also play a central role in the regulation of iron metabolism, we asked whether these two proteins are involved in diabetic iron-deficient patients. Previous studies indicated that ferrous iron (Fe^2+^) is transported across the apical membrane of enterocytes via DMT1 that mediates ferrous iron uptake. Ferrous iron must be coupled to oxidation (ferric iron) for binding to transferrin in the interstitial fluids. In the intestine, HEPH mediates iron oxidation [9]. In contrast to the effects of IDA, which usually cause significant expression of intestinal DMT1 and HEPH, we obtained no differences of these proteins among the three groups. These findings suggest the need for further investigation of the possible effect of diabetes mellitus on these two proteins.

There is evidence that iron can be stored as an intracellular iron storage protein complex consisting of heavy (H) and light (L) chain subunits (ferritins H and L). Recently, Vanoaica et al. showed that intestine-specific ferritin H deletion led to a twofold increase in iron absorption in iron-loaded mice, suggesting that ferritin H works in conjunction with systemic signals (e.g., HEPC) to limit iron flux [10]. Therefore, it was of interest to measure the ferritin L and ferritin H levels in our patients. Our data demonstrate the ferritins L and H are equally expressed in diabetic patients with or without anemia and in controls.

The normal serum ferritin (<100 ng/mL) and normal CRP levels accompanied with normal FPN among the diabetic patients without IDA are strongly against the association of IDA with chronic inflammatory state. Two limitations of our study are the small number of patients who were referred to a gastroenterologist that may represent a selection bias. In summary, we investigated DMT1, FPN, HEPH, and TfR1 expression, as well as chronic disease activity markers in diabetic patients with and without IDA and controls, demonstrated that in diabetic iron-deficient patients, this protein expression depends primarily on body iron stores rather than being affected by chronic inflammation or diabetes per se.

## Figures and Tables

**Table 1:** Demographic and clinical data of the patients.

		DM + anemia (*n* = 16)	DM (*n* = 11)	Control (*n* = 12)	*p*
Demographic	Age (years)	72 (64–78)	62 (48–69)	47 (22–71)	0.002^b,c^
	Female (%)	9 (56.3)	11 (84.6)	8 (66.7)	0.234
	Female age (years) (range)	69 (38–82)	57 (41–81)	43 (22–64)	0.017^b^
	Male age (years) (range)	73 (54–82)	69.5 (62–77)	68 (65–71)	0.152
	Duration (years)	10 (6–14)	10 (5–12)	—	0.714

Biochemistry	HbA1C (%)	6.6 (5.9–7.2)	6.8 (6.2–8.6)	5.3 (5.1–5.6)	<0.001^a,b^
	WBC (10^3^/*μ*L)	7.81 ± 2.52	6.86 ± 1.48	6.85 ± 1.44	0.367
	RBC (10^6^/*μ*L)	4.3 (3.8–4.7)	4.8 (4.4-5.1)	4.9 (4.6–5.2)	<0.001^b,c^
	HGB (g/dL)	10.9 (10.4–12.2)	13 (12.5–14.3)	14.1 (13.1–14.6)	<0.001^b,c^
	Ferritin (ng/mL)	39.2 (11.2–96.1)	60 (21–113)	51.4 (36.9–102.6)	0.756
	Glucose (mg/dL)	131.9 (105–153)	142 (112–176)	83 (81–93)	<0.001^a,b^
	Fe (*μ*g/dL)	49.1 (33.5–60.9)	84.4 (65.5–101)	65.2 (60.1–86.9)	0.001^b,c^
	Transferrin (g/L)	2.7 (2.3–3.5)	2.8 (2.6–3.2)	2.7 (2.4–2.8)	0.886
	Transferrin-sat (%)	11.6 (8.4–15.8)	22.2 (14.4–25.1)	16.6 (15.4–22.2)	0.005^b,c^
	CRP (median) (mg/L)	1.9 (1.1–8.7)	3.4 (2.3–7.4)	6.6 (1–11.9)	0.572

Medications (%)	Insulin	4 (25)	0 (0)	—	0.107
	Glyburide	3 (18.8)	1 (7.7)	—	0.606
	Metformin	7 (43.8)	7 (53.8)	—	0.588
	Sitagliptin phosphate	1 (6.3)	7 (53.8)	—	0.01
	Aspirin	7 (43.8)	9 (75)	0 (0)	<0.001^a,b^
	NSAIDs	1 (6.3)	0 (0)	0 (0)	>0.999
	PPI	15 (93.8)	7 (53.8)	2 (16.7)	<0.001^b,c^
	Iron	8 (50)	0 (0)	0 (0)	<0.001^b,c^

Comorbidities (%)	Hypertension	14 (87.5)	7 (53.8)	1 (8.3)	<0.001^a,b^
	CVD	3 (18.8)	3 (23.1)	0 (0)	0.280
	*H. pylori* positive	6 (37.5)	4 (30.8)	6 (50)	0.608

All categorical variables are presented as number and percentage, and all continuous variables are presented as median and interquartile range (IQR); DM: diabetes mellitus; NSAIDs: nonsteroidal anti-inflammatory drugs; PPI: proton pump inhibitors; CVD: cardiovascular disease: *H. pylori*: *Helicobacter pylori*. ^a^Significant *p* value between DM and control; ^b^significant *p* value between control and DM + anemia; ^c^significant *p* value between DM and DM + anemia.

**Table 2:** Comparison between iron transport proteins among the three groups.

	DM + anemia	DM	Control	*p*
Ferritin L	0.61 (0.54–1.01)	0.69 (0.34–1.07)	0.74 (0.43–0.98)	0.899
Ferritin H	0.3 (0.2–0.45)	0.43 (0.34–0.83)	0.51 (0.39–0.67)	0.085
FPN	1.17 (0.89–1.46)	0.76 (0.53–1.04)	0.71 (0.64–0.86)	0.011^b,c^
HEPH	1.03 (0.72–1.36)	0.79 (0.46–1.34)	0.85 (0.63–1.06)	0.374
DMT1	0.61 (0.42–0.89)	0.79 (0.58–0.94)	0.69 (0.52–0.84)	0.533
TfR	0.39 (0.26–0.61)	0.36 (0.24–0.43)	0.18 (0.16–0.24)	0.004^a,b^

All variables are presented as median and interquartile range (IQR). ^a^Significant *p* value between DM and control; ^b^significant *p* value between control and DM + anemia; ^c^significant *p* value between DM and DM + anemia; DM: diabetes mellitus; FPN: ferroportin; HEPH: hephaestin; DMT1: divalent metal transporter 1; TfR: transferrin receptor.
