# Corrigendum to “Expression of Duodenal Iron Transporter Proteins in Diabetic Patients with and without Iron Deficiency Anemia”

**DOI:** 10.1155/2018/1326193

**Published:** 2018-12-26

**Authors:** Efrat Broide, Ram Reifen, Shay Matalon, Zipi Berkovich, Maya Moshe, Haim Shirin

**Affiliations:** ^1^The Kamila Gonczarowski Institute of Gastroenterology, Assaf Harofeh Medical Center and Sackler School of Medicine, Tel Aviv University, Tel Aviv, Israel; ^2^School of Nutritional Sciences, Faculty of Agriculture, The Hebrew University of Jerusalem, Jerusalem, Israel

In the article titled “Expression of Duodenal Iron Transporter Proteins in Diabetic Patients with and without Iron Deficiency Anemia” [1], Dr. Maya Moshe was missing from the authors' list. Dr. Maya Moshe conducted all the laboratory work starting from protein purification to Western blotting including the analysis of the results using image processing software. They were in charge of the experimental design and the arrangement of the information from the patients' questionnaires to the tables used to divide the patients into experimental groups. They also participated in forming and interpreting the results, working on the preliminary statistics, and writing Section 2.1. The corrected authors' list is shown above.

Additionally, the “Authors' Contributions” section should be corrected to “Efrat Broide, Ram Reifen, and Maya Moshe contributed equally to this paper.”
